# MADM-based smart parking guidance algorithm

**DOI:** 10.1371/journal.pone.0188283

**Published:** 2017-12-13

**Authors:** Bo Li, Yijian Pei, Hao Wu, Dijiang Huang

**Affiliations:** 1 School of Information Science and Engineering, Yunnan University, Kunming, Yunnan, China; 2 School of Computing, Informatics, and Decision Systems Engineering, Arizona State University, Tempe, AZ, United States of America; Southwest University, CHINA

## Abstract

In smart parking environments, how to choose suitable parking facilities with various attributes to satisfy certain criteria is an important decision issue. Based on the multiple attributes decision making (MADM) theory, this study proposed a smart parking guidance algorithm by considering three representative decision factors (i.e., walk duration, parking fee, and the number of vacant parking spaces) and various preferences of drivers. In this paper, the expected number of vacant parking spaces is regarded as an important attribute to reflect the difficulty degree of finding available parking spaces, and a queueing theory-based theoretical method was proposed to estimate this expected number for candidate parking facilities with different capacities, arrival rates, and service rates. The effectiveness of the MADM-based parking guidance algorithm was investigated and compared with a blind search-based approach in comprehensive scenarios with various distributions of parking facilities, traffic intensities, and user preferences. Experimental results show that the proposed MADM-based algorithm is effective to choose suitable parking resources to satisfy users’ preferences. Furthermore, it has also been observed that this newly proposed Markov Chain-based availability attribute is more effective to represent the availability of parking spaces than the arrival rate-based availability attribute proposed in existing research.

## Introduction

Based on various sensing and communications technologies, it becomes possible to build up so-called smart parking guidance systems to monitor and collect the real time status of parking spaces in controlled areas, and then to direct drivers to available parking resources [1–8]. Such real time dynamic status, along with that static information of parking resources (e.g., the locations and capacities of the facilities), can be used for drivers to choose target parking facilities.

By regarding the status of the parking facilities as attributes and drivers’ preferences as weights, the problem to choose the most suitable parking facility can be viewed as a multiple attributes decision making (MADM) problem. Up to now, some MADM-based parking guidance algorithms with various attributes, weighting factors, and objectives have already been proposed and investigated [9–12]. Besides those static attributes of parking facilities such as physical locations, distances, and parking cost, the availability of parking resources is an important factor to determine the chance of successful parking, especially when the traffic intensity is high and the competition of available parking spaces is intensified.

In existing research, the availability of parking resources is often defined as some kind of probabilities of estimated numbers of vacant parking spaces as in [13], some occupancy ratio of the number of spaces currently vacant or occupied to the capacity of the facility as in [14], or the ratio of the number of expected number of newly arriving vehicles to the number of currently vacant spaces as in [15].

Many methods have already been proposed to predict the availability of parking resources based on queueing models [16–18], neural networks [14, 19], time series models [20], multivariate autoregressive model [13], or some other simpler methods as presented in [15]. Even though those methods are reported effective to predict the availability of parking resources, when those resultant availability values in [13–15] are adopted directly as the availability attribute in MADM-based algorithms to choose the most appropriate parking facility among more than one parking facilities, they are not as effective as expected.

In this study, the root cause of why these seemly reasonable availability results as in [15] and [13] are non-effective in MADM-based parking guidance algorithms was identified: compared with other attributes such as parking distances, walking distances, or parking cost, the expected number of vacant parking spaces, rather than various probabilities or ratios related to the number of parking spaces, will be an effective attribute to determine in which candidate parking facility it is easier to find available parking spaces.

To verify the effectiveness of the expected number of vacant parking spaces as an important attribute in MADM-based parking guidance algorithms, this paper proposes a framework to implement such an MADM-based algorithm by considering three typical attributes of candidate parking facilities, including the estimated round-trip walking distance, the parking fee, and the availability degree of vacant parking spaces. By assigning different weights to those attributes, six typical parking preferences (i.e., Pref-I, Pref-II, to Pref-VI) were made for the MADM-based algorithm.

Especially, to predict the expected number of vacant parking spaces, based on queueing theory, for any parking facility with capacity *c*, it is modeled as an *M*/*M*/*c*/*c* queueing system, and the dynamical change of the number of vacant parking spaces is modeled as a Markov chain. In this way, the transition probability of finding *k*(≥ 0, ≤ *c*) vacant parking spaces in a future time interval can be calculated in closed form. Then, the expected number of vacant parking spaces is defined as the sum of the products of every possible number of vacant spaces and its corresponding transition probability. This new Markov Chain-based availability definition is different from the arrival rate-based availability definition proposed in [15]. Based on these two availability definitions, two different availability attributes were constructed and applied for availability-related preferences.

For drivers without any information of parking facilities, blind search is an intuitive approach to find available parking spaces by cruising on roads blindly until some vacant parking spaces appear. In this study, an enhanced self-avoiding blind search (SABS) approach was adopted as the baseline parking approach.

A simulation platform was built up to investigate the effectiveness of the SABS approach and the MADM-based algorithm in comprehensive scenarios with various distributions of parking facilities, traffic intensities, and user preferences. Their performance was measured and compared in terms of average parking failure rate, average walking distance, average driving distance, and average parking fee.

Experimental results show that every preference with the Markov Chain-based availability attribute can always choose the appropriate parking facility to satisfy the emphasized assignment, indicating that the proposed parking guidance framework and the MADM-based algorithm are effective to help drivers with various preferences find proper parking resources. Furthermore, it has also been proved that the Markov Chain-based availability attribute is more effective to represent the availability of parking spaces than the arrival rate-based attribute proposed in [15].

To summarize, the main novel contributions of this paper are:

- Identify and define the expected number of vacant parking spaces as the core attribute to reflect the difficulty of finding available parking spaces, and proposed a queueing-theory based method to estimate the expected number of vacant spaces;
- Proposed an MADM-based parking guidance algorithm for choosing one appropriate parking facility among a group of candidates, by considering three representative decision factors and various preferences of drivers.
- Investigated the effectiveness of the MADM-based algorithm against the baseline SABS algorithm in comprehensive scenarios with various distributions of parking facilities, traffic intensities, and user preferences. Both the Markov Chain-based availability attribute and the arrival rate-based availability attribute were considered for availability-related preferences.

The remainder of this paper is structured as follows: the Section on Related Work conducts a brief analysis of existing research on smart parking guidance systems; Section MADM-based Parking Guidance Framework presents the framework to implement such an MADM-based parking guidance system. Then, the MADM-based Parking Guidance Algorithm Section and the Self-avoiding Blind Search Algorithm(SABS) Section present the details of the MADM-based algorithm and the SABS strategy individually. In Section Simulations and Results, simulations for six typical preferences of the MADM-based algorithm were carried out and their effectiveness has been proved and compared with the SABS strategy with regards to the average parking failure rate, the average walking distance, and the average parking cost. The reason why those methods adopted in existing research are not effective to reflect the availability degree of vacant parking spaces is discussed in the Discussion section. Finally, we summarized the work of this paper in the Conclusion section.

## Related work

In smart parking guidance systems, many factors, including the availability of parking spaces, parking price, driving distance to the guided parking facility, walking distance from the guided parking facility to the destination, etc., can be considered in selecting parking facilities [15, 21, 22]. Among those factors, the availability of parking spaces is very important, and some methods have already been proposed to predict the availability of parking resources on the basis of queueing models [16–18], neural networks [14, 19], time series models [20], multivariate autoregressive model [13], or some other simpler methods as presented in [15]. Technologically speaking, those methods may be different, but, all of them were reported to be effective to estimate the availability of parking resources at certain acceptable degree of accuracy.

From the perspective of the methods for estimating the availability of parking spaces, the queueing models and solutions in [16–18] are closely related to ours. In [16–18], smart parking facilities are viewed as queueing systems, and a continuous-time Markov model is built to predict parking lot occupancy in vehicular ad hoc networks. In [16], a parking lot is modeled as an *M*/*G*/*c*/*c* system, and the blocking probability that all parking spaces are occupied is presented, then the parking lot’s capacity and its blocking probability can be disseminated to help drivers choose parking facilities. In [17], a parking lot is represented as a homogeneous Markov model with exponentially distributed inter-arrival and parking times, and an *M*/*M*/*m*/*m* model is used to predict the probability *p*_*ij*_ that the number of vehicles parking on the parking lot is *j* (i.e., the state of the Markov chain is in state *j*) at *t* time units in the future, given that its present state is *i*. In [18], the same model as in [17] was used to represent parking lots and a method was proposed to calculate the matrix exponential operator in *p*_*ij*_. In our paper, a parking facility is modeled as an *M*/*M*/*c*/*c* queueing system, which is identical to the adopted *M*/*M*/*m*/*m* model in [17, 18] except that the number of parking spaces is represented as *c*, instead of *m*, and then, the expected number of vacant parking spaces is calculated and used as the availability attribute of the MADM-based algorithm.

Based on those predicting methods, estimation results of the availability of parking spaces can be derived and used by parking guidance algorithms. In existing research, the availability of parking resources is often defined as some kind of probabilities of estimated numbers of vacant parking spaces as in [13], some occupancy ratio of the number of spaces currently vacant or occupied to the capacity of the facility as in [14], or the ratio of the number of expected number of newly arriving vehicles to the number of currently vacant spaces as in [15]. Intuitively, these definitions can be used directly as the availability attribute in MADM-based algorithms. However, they are as effective as expected. For example, in [15], the authors proposed a method to quantify the degree of available parking spaces by defining it as the ratio of the expected number of newly arriving vehicles to the number of currently vacant parking spaces. Then, this definition was used directly as the availability attribute in the proposed MADM-based parking guidance algorithm. However, as concluded by the authors from the simulation results, in one preference originally designed to focus on finding parking spaces with higher availability and reducing parking failure rate, it seems that *“the preferences used in the assignment do not have much effect on the parking failure rate”*. This conclusion pronounces the availability attribute adopted in [15] is not effective to find parking facilities with more vacant parking spaces.

A second example, in [13], the highest probability of having at least one available spot to the driver is proposed as a criterion to recommend parking locations. When this highest probability was used as the availability attribute in our MADM-based algorithm to choose parking facilities, it was shown in simulations that this attribute has very little influence on choosing parking facilities, no matter what its weight is.

In our paper, the root cause why those availability definitions in existing research are not effective in MADM-based algorithms is identified, and the expected number of vacant parking spaces is regarded as an important attribute for the proposed MADM-based algorithm. Based on queueing theory, for any parking facility with capacity *c*, it is modeled as an *M*/*M*/*c*/*c* queueing system, and the dynamical change of the number of vacant parking spaces is modeled as a Markov chain. Then, the expected number of vacant parking spaces is calculated as the sum of the products of every possible number of vacant spaces and its corresponding transition probability. The effectiveness of this newly defined attribute, along with the MADM-based parking guidance algorithm, was investigated and proved via simulations.

## MADM-based parking guidance framework

**Fig 1:**
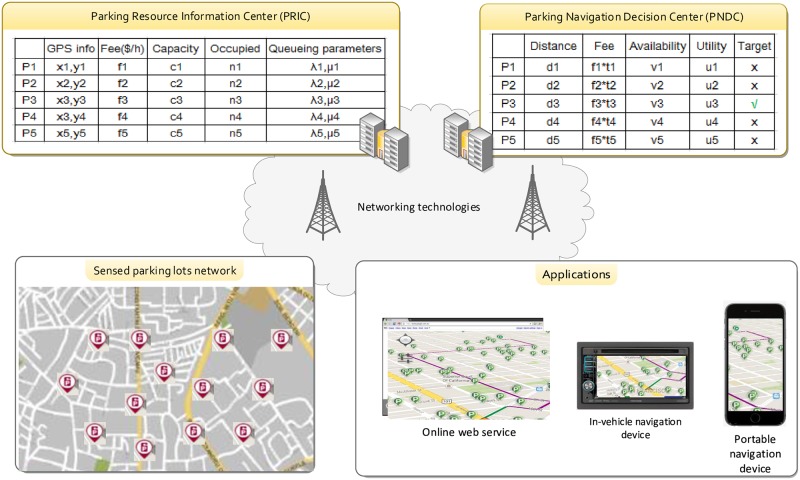
Framework of the online realtime smart parking guidance system.

Fig 1 illustrates the overall architecture of the smart parking guidance system. Assume the parking facilities within the *sensed parking facilities network* are equipped with various sensing and communication devices to monitor and disseminate the status of the facilities. Many sensing devices, such as video and image signal processors, microwave radar, ultrasonic sensors, and radio-frequency identification (RFID) readers, can be used to monitor the status of parking spaces. The sensors within the same parking facilities can be connected to a local server, which will relay the status information to remote PRIC and PNDC via wired or wireless communication links. PRIC and PNDC could be a remote cloud server running by public administrators or commercial service providers. In [23], various enabling technologies for smart parking guidance systems were reviewed.

Then, for every parking facility, its attributes, ranging from its GPS location, parking fee rate, the maximum amount(i.e., capacity) of parking facilities, the number of occupied spaces, to the arrival and leaving process of the vehicles, are transferred to the *Parking Resource Information Center* (PRIC). Users may access PRIC online to get the distributions and attributes of the parking facilities within given areas.

Furthermore, such information stored in PRIC can also be used to generate real time parking navigation for users in moving vehicles. This can be implemented by using the centralized *Parking Navigation Decision Center* (PNDC) or by using distributed in-vehicle or portable navigation devices. The core difference between the two implementations lies on which entity is responsible for making the navigation decision. For the centralized structure, before arriving the destination, users’ navigation device will send parking requests to PNDC, and the latter will access the information of the parking facilities stored in PRIC and choose suitable spaces for the users according to certain criteria, such as the distance from the destination, parking fee, or the availability of parking spaces. For example, in Fig 1, P3 is selected as the target parking facility to which the vehicle will be guided. For the distributed structure, users’ navigation device will access the information stored in PRIC directly and choose suitable parking spaces by themselves in the same way used by PNDC.

## MADM-based parking guidance algorithm

**Fig 2:**
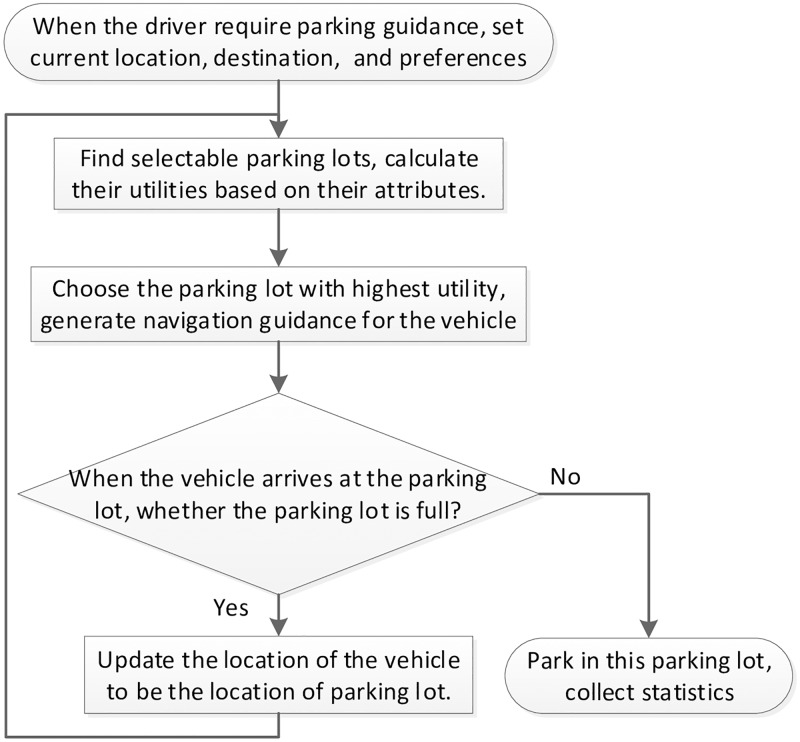
Diagram of the MADM-based parking guidance algorithm.

The smart parking navigation problem can be regarded as how to choose appropriate parking facilities for the drivers according to some criteria, and then guide them from their current location to the chosen park facilities. On condition that the locations of the vehicles and the parking facilities are known, assume the shortest path routing algorithm can be used to guide the drivers from one location to others. So, in this paper, we mainly focus on the problem of choosing appropriate parking facility. In reality, this problem is about how to select some parking resources to satisfy certain criteria among a limited number of candidates with various attributes (such as the distance from the destination, parking fee, and the availability of finding vacant spaces). This is a typical multiple attributes decision-making (MADM) problem. Fig 2 depicts the diagram of the MADM-based parking guidance algorithm.

For a user, assume her/his current location is *P*_*s*_, the driving destination is *P*_*d*_, and there are *n*(> 0) parking facilities, identified as *P*_1_, *P*_2_, …, and *P*_*n*_ individually, that are available for the user. Such a parking environment can be modeled as a directed or undirected graph, in which current location, the destination, and the parking facilities are nodes, and the roads that directly connect some pairs of nodes are edges. If all paths are open for two-way traffic, the graph is undirected; otherwise, the edges corresponding to those one-way paths are directed. For parking facility *P*_*i*_(1 ≤ *i* ≤ *n*), assume its properties can be expressed by such parameters as its GPS coordination (*x*_*i*_, *y*_*i*_), parking fee rate *f*_*i*_, capacity *C*_*i*_, the number of occupied spaces *n*_*i*_, the arrival rate λ_*i*_ and the service rate *μ*_*i*_.

### MADM problem description

Assume there are *n* candidate parking facilities *P* = {*P*_1_, *P*_2_, …, *P*_*n*_}, and for *P*_*i*_(1 ≤ *i* ≤ *n*), there are *m* attributes. The MADM decision matrix *D* and its normalized matrix $\bar{D}$ can be expressed as follows:

$$\begin{array}{cccccccccccccc} & & & a_{1} & a_{2} & \cdots & a_{m} & & & & a_{1} & a_{2} & \cdots & a_{m}\\ D & = & \begin{array}{l} P_{1}\\ P_{2}\\ \vdots \\ P_{n} \end{array} & (\begin{array}{c} a_{11}\\ a_{21}\\ a_{n1} \end{array} & \begin{array}{c} a_{12}\\ a_{22}\\ \vdots \\ a_{n2} \end{array} & \begin{array}{c} \cdots \\ \cdots \\ \cdots \\ \cdots \end{array} & \begin{array}{c} a_{1m}\\ a_{1m}\\ a_{1m} \end{array}), & \bar{D} & = & \begin{array}{l} P_{1}\\ P_{2}\\ \vdots \\ P_{n} \end{array} & (\begin{array}{c} \bar{a_{11}}\\ \bar{a_{21}}\\ \bar{a_{n1}} \end{array} & \begin{array}{c} \bar{a_{12}}\\ \bar{a_{22}}\\ \vdots \\ \bar{a_{n2}} \end{array} & \begin{array}{c} \cdots \\ \cdots \\ \cdots \\ \cdots \end{array} & \begin{array}{c} \bar{a_{1m}}\\ \bar{a_{1m}}\\ \bar{a_{1m}} \end{array}) \end{array}$$

where *a*_*ij*_ denotes the *jth* attribute of *P*_*i*_, and $\bar{a_{ij}}$ denotes the normalized value of *a*_*ij*_. The normalization of the values is performed according to two situations(the-larger-the-better, the smaller-the-better) as follows:

$$\begin{array}{c} \bar{a_{ij}}=\left(a_{j}^{max}-a_{ij}\right)/\left(a_{j}^{max}-a_{j}^{min}\right) \end{array}$$ (1)

$$\begin{array}{c} \bar{a_{ij}}=\left(a_{ij}-a_{j}^{min}\right)/\left(a_{j}^{max}-a_{j}^{min}\right) \end{array}$$ (2)

where $a_{j}^{max}$ and $a_{j}^{min}$ are the maximum and the minimum of attribute *a*_*j*_ respectively.

Simple Additive Weighting (SAW) is an effective method to calculate the overall utility of a candidate parking facility, which is determined by the weighted sum of all the attribute values. With given attribute values, there are many ways to determine the weights. Assume drivers may have different preferences about these attributes. To reflect such preference, weight *w*_*j*_ can be used to denote the percentage of importance of the *jth* attribute. Provided the decision matrix and associated weights are known, define the utility of *P*_*i*_(*i* = 1, 2, …, *n*) as:

$$\begin{array}{c} U_{i}=\sum_{j=1}^{m}w_{j}\bar{a_{ij}} \end{array}$$ (3)

Finally, since a larger utility value leads to better performance, the parking facility with the maximum utility is selected as the target, and the navigation device will guide the driver to the selected parking facility with detailed information.

In this paper, three key attributes are taken into account:

- *a*_*i*1_: estimated round-trip walking distance from *P*_*i*_ to the destination of the driver;
- *a*_*i*2_: estimated parking fee, = *f*_*i*_ × (*t*_*p*_ + *a*_*i*1_), where *t*_*p*_ is the estimated stay time of the driver at the destination. By adding *a*_*i*1_ to *t*_*p*_, we get the total parking time.
- *a*_*i*3_: estimated availability degree of vacant parking spaces at *P*_*i*_ when the vehicle arrives.

According to the normalization process defined in Eqs (1) and (2), *a*_*i*1_ and *a*_*i*2_ should be normalized by Eq (2), and *a*_*i*3_ should be normalized by Eq (1), in that a smaller walk duration or parking fee, or a larger probability of finding vacant space will lead to better performance.

For each *P*_*i*_(*i* = 1, 2, …, *n*), its attributes *a*_*i*1_, *a*_*i*2_, and *a*_*i*3_ can be easily estimated by taking into account its physical distances from *P*_*d*_, walk speed, the parking fee rate, and the estimated parking duration. However, it is a challenge to estimate the availability of vacant parking spaces in each parking facility. In the following section, a queueing model is proposed for estimating the availability of parking spaces.

### Queueing model for estimating the availability degree

At time *t*_0_ when a user sends request to PNDC or PRIC for parking guidance information, assume PNDC or PRIC can collect the real time status of every parking facility *P*_*i*_(*i* = 1, 2, …, *n*), including the capacity *c*_*i*_, the number of vacant spaces *n*_*i*_, and the number of occupied spaces *c*_*i*_ − *n*_*i*_. Furthermore, assume the arrival and the leaving of vehicles in *P*_*i*_ follow Poisson distributions with parameters λ_*i*_ and *μ*_*i*_ respectively, we can model the dynamic behavior of every parking facility *P*_*i*_ as an *M*/*M*/*c*/*c* queueing problem in Kendall’s notation [24], where the first and second *M* mean the arrival and leaving of vehicles follow Poisson distribution, the first *c* means the number of parking spaces, and the second *c* means the capacity of the queue. If there is no vacant space available, assume users are not allowed to stay and circulate in the parking facility to wait for the leaving of parked vehicles.

**Fig 3:**
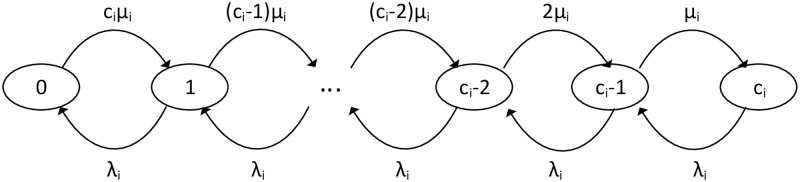
Markov chain model for parking facility *P*_*i*_ with capacity *c*_*i*_. The states correspond to the number of vacant spaces, λ_*i*_, the arrival rate of vehicles, and *μ*_*i*_, the service rate.

For the *M*/*M*/*c*/*c* queueing model with parameters λ_*i*_ and *μ*_*i*_, providing the state *x*(*t*)(= 0, 1, 2, …, *c*) be the number of vacant stalls in the facility at time *t*(> 0), and the driving duration from current location to the parking facility is *τ*(> 0), the problem to estimate available spaces at *t* + *τ* can be expressed as a continuous time discrete state Markov process (Fig 3): at time *t*, the number of vacant spaces(i.e., the state of the queue) is *s*_*i*_; at time *t* + *τ*, the probability of finding *s*_*j*_ vacant spaces is the transition probability *p*_*s*_*i*_*s*_*j*__(*t*, *t* + *τ*). For simplicity, assume the process is time homogeneous, i.e., its transition probability is independent to the start time *t*, we have *p*_*s*_*i*_*s*_*j*__(*t*, *t* + *τ*) = *p*_*s*_*i*_*s*_*j*__(*τ*).

According to the results of *M*/*M*/*c*/*c* queueing model [17, 18, 24], we have

$$\begin{array}{c} p_{s_{i}s_{j}}\left(\tau \right)=e^{Q\tau }. \end{array}$$ (4)

where *Q* is the one-step transition probability matrix that can be expressed as:

$$\begin{array}{c} Q=\left[\begin{array}{c} \begin{array}{ccccc} \overset{-c\mu }{\lambda_{i}} & \overset{c\mu }{\underset{\lambda_{i}}{-(\lambda_{i}+(c-1)\mu_{i})}} & \underset{-(\lambda_{i}+(c-2)\mu_{i})}{(c-1)\mu_{i}} & \underset{(c-2)\mu_{i}}{} & \\ & & \vdots & & \\ & & \lambda_{i} & \underset{\lambda_{i}}{-(\lambda_{i}+\mu_{i})} & \underset{-\lambda_{i}}{\mu_{i}} \end{array} \end{array}\right] \end{array}$$ (5)

Let *π*_*s*_*i*__ be the vector of length *c* + 1 to represent the initial probability distribution of the states at *t*, in which the value at the *kth* position is the probability for state *k* − 1. At time *t*, since the number of vacant spaces is *s*_*i*_, only the *s*_*i*_ + 1 entry of *π*(*t*) is 1, and all the other entries are 0. The probability distribution of state *s*_*j*_ at time *t* + *τ* can be expressed as:

$$\begin{array}{c} \pi_{s_{j}}\left(\tau \right)=\pi_{s_{i}}p_{s_{i}s_{j}}\left(\tau \right). \end{array}$$ (6)

For the solution of *π*_*s*_*j*__(*τ*), the value in its *jth* entry represents the probability of finding *s*_*j*_ − 1 vacant stalls at time *t* + *τ*, and the entry with the maximum probability indicates the most possible number of vacant parking spaces. The value in the first entry is the probability of finding zero vacant parking space, which can be defined as the blocking probability *π*_0_(*τ*). So, the probability of finding at least one vacant parking space is

$$\begin{array}{c} \pi_{s_{j}>0}\left(\tau \right)=1-\pi_{0}\left(\tau \right). \end{array}$$ (7)

Intuitively, *π*_*s*_*j*_>0_(*τ*) can be used to describe the availability degree of parking facilities. However, for a group of candidate parking facilities with various capacity, initial status, or *τ*, this probability can not represent the difficulty degree of finding vacant spaces in each facility, just as what happens in [15]. For example, assume the values of *π*_*s*_*j*__(*τ*) of two parking facilities *P*_1_ and *P*_2_ with capacity 5 are listed in Table 1. Although the blocking probabilities of them are the same 0.1 and their probabilities of finding at least one parking spaces are the same 0.9, the difficulty degree of finding vacant parking spaces are quite different: for *P*_1_, the expected number of vacant parking spaces is 1.9; but for *P*_2_, the expected number is as high as 3.1. Obviously, for current vehicle, it will be more difficult to find a vacant parking space in *P*_1_ than in *P*_2_. So, in this paper, for parking facility *P*_*i*_ with capacity *c* and initial status *s*_*i*_, the expected number of vacant parking spaces is defined as the availability attribute (i.e., *a*_*i*3_) to describe the availability degree of vacant parking spaces.

**Table 1:** Example transition probabilities and the availability of vacant parking spaces of two parking facilities *P*_1_ and *P*_2_ with capacity 5.

	0	1	2	3	4	5	Availability
*P*_1_	0.1	0.4	0.2	0.15	0.1	0.05	**1.9**
*P*_2_	0.1	0.1	0.1	0.15	0.4	0.15	**3.1**

$$\begin{array}{c} availability=\sum_{s_{j}=0}^{c}s_{j}*p_{s_{i}s_{j}}\left(\tau \right) \end{array}$$ (8)

Based on above analyses, we can estimate the utilities of candidate parking facilities by using Eq (3) and select the one with the maximum utility. When a guided smart vehicle arrives at the target parking facility, if the parking facility is not fully occupied, the vehicle will be allowed to park. Otherwise, a new guiding process will be triggered to find a new target parking facility.

The effectiveness of this definition adopted here to describe the availability degree of parking resources will be investigated via comprehensive simulations in Section Simulations and Results. For the comparison of the effectiveness of the proposed availability definition, the arrival rate-based availability definition proposed in [15] is also considered in all MADM-based parking guidance simulations. Furthermore, in order to compare the overall effectiveness of these MADM-based parking guidance strategies, one *blind search*-based parking guidance strategy is used as the baseline parking strategy.

## Arrival rate-based availability definition

In [15], the degree of availability for the parking facility *j* is defined as:

$$\begin{array}{c} R_{ij}=\frac{T_{ij}/MTBA_{j}}{f_{j}} \end{array}$$ (9)

where *T*_*ij*_ is the estimated time for a guided vehicle to drive from current location to the target parking facility, *MTBA*_*j*_ the mean time between car arrivals of parking facility *j*, and *f*_*j*_ the number of currently vacant parking spaces.

The lower value of *R*_*ij*_ is assumed to indicate that “it is more likely to find the free parking facility when a driver arrives at parking facility *j* since fewer cars are expected to come compared to the number of free parking facilities”. When this definition is used as the availability attribute in the MADM-based parking guidance algorithm, it is normalized by using Eq (2).

To distinguish the availability definition proposed in [15] and the one proposed in our paper, in following sections, the former is name as the arrival rate-based definition, and the latter the Markov Chain-based definition.

## Self-avoiding blind search algorithm(SABS)

For the comparison of the proposed MADM-based parking guidance algorithm, another *blind search* strategy has also been considered in this paper. In this strategy, we assume the locations and the status of the parking facilities are not available for the vehicles, thus the drivers who want to park have to circulate blindly in the streets until an available parking space is found.

In [15], it is assumed that every vehicle knows the location of the nearest parking facility. In a more realistic environment, we can assume all the drivers without navigation device do not know any information about the parking facilities. In this case, when they want to park, they have to circulate blindly in the streets to find available parking spaces.

In mathematics, many random walk strategies can be adopted by the drivers to find parking facilities. A self-avoiding random walk is a sequence of moves on a lattice that does not visit the same point more than once. It is reported in [25] that self-avoiding random walk is the best search strategy in complex networks to find a target node. So, in this study, we assume the drivers who are looking for available parking facilities blindly will adopt a similar self-avoiding random walk strategy.

**Fig 4:**
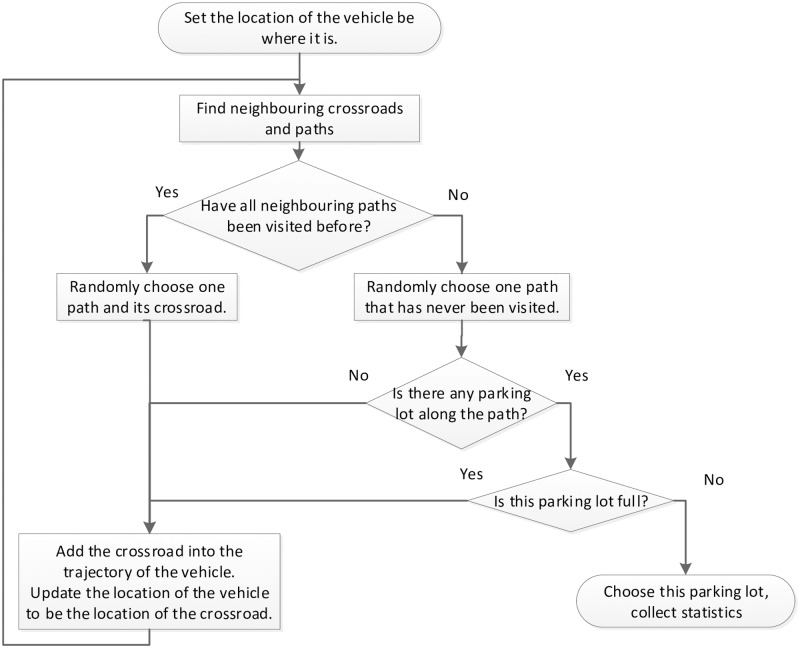
Diagram of the base self-avoiding blind search (SABS) strategy.

Fig 4 depicts the diagram of the strategy in detail. For a vehicle, at the beginning, its location is set as where it is. Then, all neighboring crossroads, as well as the paths leading to those crossroads, are figured out. Assume the driver can always remember the paths that have already been visited. If all the selectable paths have been visited before, the driver has to choose one path randomly, move to the crossroad at the end of the path, add the crossroad into its trajectory, update its location, and begin a new round of search. Otherwise, the driver will randomly choose one path among those that have never been visited yet. If there is any parking facility along the path and it is not full, the vehicle will take a parking space. In any other situations, the vehicle will move to the end crossroad of the path and begin a new round of search.

Different from the original self-avoiding random walk strategy in complex networks in [25] that tries to avoid revisiting nodes twice, in the self-avoiding random walk strategy of this study, vehicles are allowed to revisit the same crossroad more than once, on condition that they try to avoid those paths that have already been visited. Such a relaxed condition may be useful for the vehicles to avoid being trapped in a dead end.

**Fig 5:**
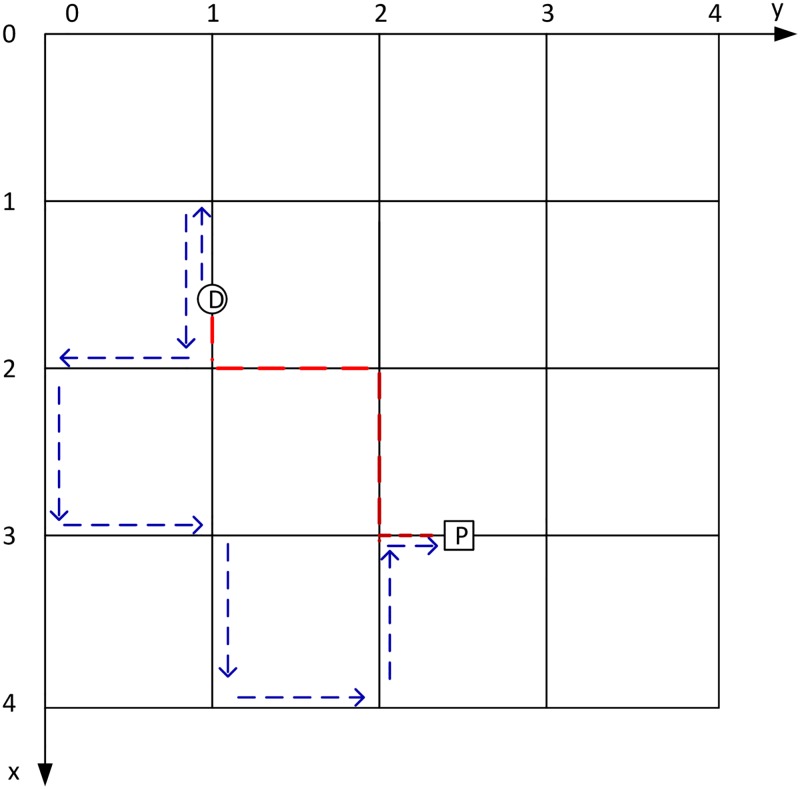
Example of the self-avoiding blind search (SABS) strategy.

Fig 5 illustrates an example of the SABS parking strategy in a parking area with squared street blocks. The blue lines constitute an example driving route from the destination D to the parking facility P made based on SABS, and the red lines constitute one of the shortest driving or walking routes that may be adopted by other parking preferences in which the locations of the parking facilities are known.

## Simulations and results

### Simulation setup

**Fig 6:**
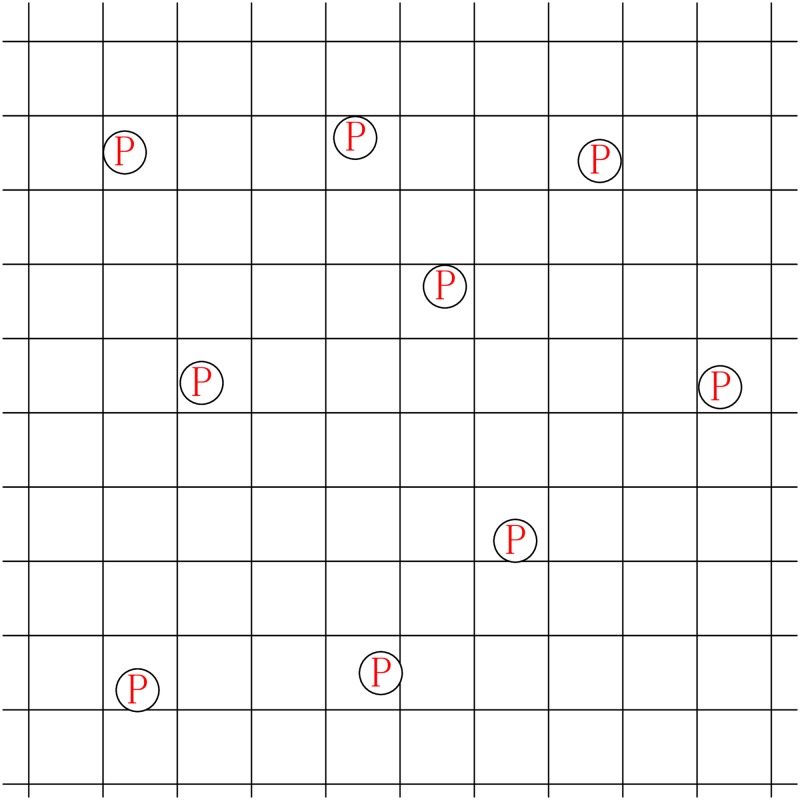
Simulation area (1 × 1 km) with 100 × 100 m square blocks.

Simulations were carried out to evaluate the effectiveness of the proposed MADM-based parking guidance algorithm, as well as the arrival rate-based availability definition proposed in [15] and the Markov chain-based definition proposed in our paper. A 1 × 1 km area with 100 × 100 m square blocks in downtown Houston, Texas is used as the simulation area (see Fig 6). Within this area, there are some parking facilities scattered in different blocks, and some vehicles with or without navigation devices are looking for parking spaces.

To support the simulation of different parking environments, Table 2 lists all the control parameters that can be used for generating different simulation scenarios, including the density of parking facilities within the area, the attributes of each parking facility, the arrival and leaving process of vehicles in each parking facility.

**Table 2:** Control parameters for generating parking facilities and vehicles.

Parameter	Definition	Value scope
*α*	parking facility density	0.1
*β*	smart vehicle ratio	0.05
*c*_*i*_	capacity of *P*_*i*_	*U*(30, 150), step size = 1
*f*_*i*_	parking fee rate	*U*(0.25, 1) $ per 15 min, step size = $0.25
*μ*_*i*_	service rate	1/*μ*_*i*_ = 51 min, service time follows *Exp*(1/*μ*_*i*_)
*ρ*_*i*_	traffic intensity	low intensity, *U*(0.4, 1), expectation = 0.7
		medium intensity, *U*(0.7, 1.3), expectation = 1
		high intensity, *U*(1, 1.6), expectation = 1.3
λ_*i*_	arrival rate	λ_*i*_ = *ρ*_*i*_*c*_*i*_*μ*_*i*_, inter-arrival time follows *Exp*(1/λ_*i*_)

Assume there is at most one parking facility in one block. The density *α* of parking facilities is defined as the ratio of the number of parking facilities to the number of blocks. For each parking facility, assume there is only one entrance/exit randomly located on the perimeter of the block, the location of which is regarded as that of the parking facility.

For each parking facility, the time duration that vehicles will stay in the parking facility is determined by the service rate *μ*. In [17], it is reported that the expected parking time *μ*^−1^ is 51 minutes. Therefore, in our simulations, 51 minutes is regarded as a standardized time unit to express all time-related parameters, i.e., one time unit in simulation corresponds to 51 minutes in reality. For parking facility *P*_*i*_ with capacity *c*_*i*_ and service rate *μ*_*i*_, its traffic intensity *ρ*_*i*_(= λ_*i*_/*c*_*i*_ *μ*_*i*_) is defined to reflect the degree of occupancy. As the value of *ρ*_*i*_ increases, the occupancy degree will be higher, and it will be more difficult to find vacant spaces. On condition that the values of *ρ*_*i*_, *μ*_*i*_, and *c*_*i*_ are known, the value of arrival rate λ_*i*_(= *c*_*i*_ *μ*_*i*_ *ρ*_*i*_) can be figured out, which is used to control the arrival of vehicles that want to park in parking facility *P*_*i*_. According to the values of λ_*i*_ and *μ*_*i*_, a group of vehicles can be generated for parking facility *P*_*i*_.

Among all the vehicles, assume the proportions of vehicles equipped with and without navigation devices are *β* and 1 − *β* individually. For those vehicles without navigation devices, they will arrive at their specified parking facilities at given time points. At that moment, if the specified parking facility is full, the vehicle will be rejected immediately. Otherwise, it will be allocated with a vacant space and allowed to stay for a given duration specified by its service time. For those vehicles with navigation devices, assume their destinations are randomly distributed along the streets. The drivers will require parking guidance when they move close to their destination, then such requirements will be processed locally by the navigation devices or be transferred to PNDC to process remotely. No matter how the requirements are processed, target parking facilities will finally be selected for the drivers according to their preferences. When a guided vehicle arrives at a parking facility, if the parking facility is not full, the vehicle will be allocated with a vacant space and allowed to stay for a given time interval specified by its service time. Otherwise, it will be rejected. In such case, the driver will require another parking guidance until he/she finally finds out an available parking space.

### Preferences and performance metrics

Similar to the user preferences used in [15], in this study, we also set different combinations of weight values to represent different user preferences. As mentioned before, for the MADM-based parking guidance algorithm, three key attributes, i.e., the estimated round-trip walking distance (*a*_*i*1_), the parking fee (*a*_*i*2_), and the availability degree of vacant parking spaces (*a*_*i*3_), were taken into account in this paper. By assigning different weights to those attributes, different parking preferences can be made. Table 3 lists six representative preferences for the MADM-based parking guidance algorithm with different weight combinations.

**Table 3:** Representative preferences of the MADM-based parking guidance algorithm.

Preference	Attributes	Combination of weights	Description
I	{*a*_*i*1_, *a*_*i*2_, *a*_*i*3_}	{1, 0, 0}	Guide based on known locations
II	{*a*_*i*1_, *a*_*i*2_, *a*_*i*3_}	{0.5, 0.5, 0}	Guide based on known locations and parking fees
III	{*a*_*i*1_, *a*_*i*2_, *a*_*i*3_}	{0.6, 0.2, 0.2}	Emphasize on shorter round-trip walking distance from a parking facility to the destination
IV	{*a*_*i*1_, *a*_*i*2_, *a*_*i*3_}	{0.2, 0.6, 0.2}	Emphasize on lower parking fee
V	{*a*_*i*1_, *a*_*i*2_, *a*_*i*3_}	{0.2, 0.2, 0.6}	Emphasize on higher availability of parking spaces
VI	{*a*_*i*1_, *a*_*i*2_, *a*_*i*3_}	{1/3, 1/3, 1/3}	Equally emphasize on all three attributes.

In existing navigation software, drivers usually can access some static information of surrounding parking facilities, e.g., locations and parking fee rates. In Table 3, Preferences I and II are designed for these cases: for Preference I, assume only the locations of parking facilities are known to drivers, and this information can help them find nearby parking facilities by avoiding blind search; For Preference II, assume both the locations and parking fee rates of parking facilities are known, and drivers can make parking decisions based on these two factors. For Preferences III to VI, the availability degree of parking spaces is taken into account.

To evaluate the effectiveness of the base SABS algorithm and the MADM-based parking guidance algorithm with various preferences on guided vehicles, following measures were considered:

- Average failure rate: when a driver arrives at a full parking facility, he/she will be rejected. This measure is defined as the total number of rejections happened to guided vehicles divided by the total number of guided vehicles.
- Average driving distance: this measure indicates the average driving distance for guided vehicles to find available parking space from where the drivers firstly send out their parking requirements. It is calculated as the sum of all driving distances of guided vehicles divided by the number of guided vehicles. The less value of this measure also means shorter driving time, less fuel consumption, and less pollution emission in the process of finding available parking spaces.
- Average walking distance: this metric is used to evaluate how far it is for drivers to walk from their destinations to selected parking facilities. Assume every driver always knows how to find the shortest walk path from the selected parking facilities to his or her destination, and always follows such shortest walk path. The average walking distance is calculated as the sum of round-trip walk distances of all guided drivers divided by the number of them. Usually, drivers prefer shorter walking distance to reduce walking time.
- Average parking fee: this metric is used to evaluate the average amount of money paid for each parking. For each guided vehicle, its parking fee is calculated as the product of the parking fee rate of the selected parking facility and the duration that the vehicle will stay. The average parking fee is the total amount of parking fee paid by guided drivers divided by the number of them.

### Simulation results and analysis

To investigate the performances of the base SABS algorithm and the MADM-based algorithm with various user preferences and parking scenarios, we have developed an event-driven simulation platform in Matlab to model the attributes and behaviors of the components in the smart parking guidance environment illustrated in Fig 1. Different parking scenarios were generated, and the SABS algorithm and the MADM-based algorithm were used for smart vehicles to find appropriate parking facilities. Furthermore, in the MADM-based algorithm, in order to distinguish the impact of the arrival rate-based definition proposed in [15] and the Markov Chain-based definition proposed in our paper, both two definitions were used individually for same parking scenarios.

Before running simulations, we need to initialize the simulation environment by generating concrete parking facilities and vehicles. This is implemented as follows: at first, set the values of the parameters listed in Table 2; then, a set of parking facilities with concrete attributes will be generated within the simulation area. At the same, for each parking facility, the values of traffic intensity(*ρ*_*i*_), arrival rate (λ_*i*_), service rate (*μ*_*i*_), and smart vehicle ratio (*β*) will also be known. According to such values, vehicles with and without navigation devices will be generated.

At the beginning stage, assume the parking facilities are not empty, i.e., there are some vehicles that have already been parked in these parking facilities with known service times. For each parking facility, the number of parked vehicles at the beginning of the simulation is set randomly according to its steady state probability. The process to calculate the steady state probability is presented in Section Appendix.

After initialization, we can run the simulation by modeling the activities of drivers and vehicles according to the ascending order of their arrival time points. In this process, different strategies and user preferences may be applied for the vehicles to find available parking spaces. The behaviors of each vehicle are recorded for further analysis. When all the vehicles generated have already been processed, the simulator will stop, and we can find out the results of the performance metrics.

To investigate the performance of parking strategies in various parking environments statistically, for each set of simulation parameters and preferences, 10 rounds of simulations were carried out and their average results were used to reflect the performance of the strategies in specified parking environment, as illustrated in following subsections. In each round of simulation, the number of vehicles equipped with parking guidance devices was 1500, and the average resulting values of those vehicles were regarded as those of current round of simulation, with a 95% confidence level.

**Fig 7:**
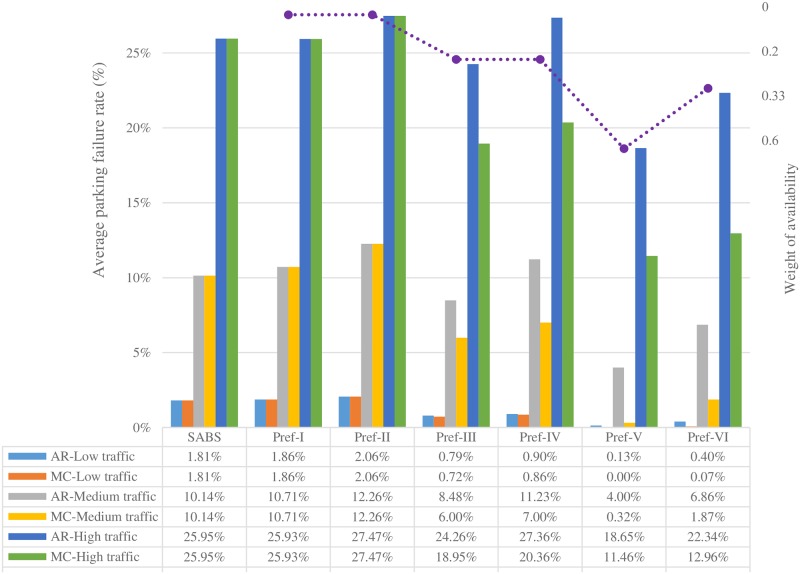
Average parking failure rate results of the strategies with different availability attributes and parking traffic.

**Fig 8:**
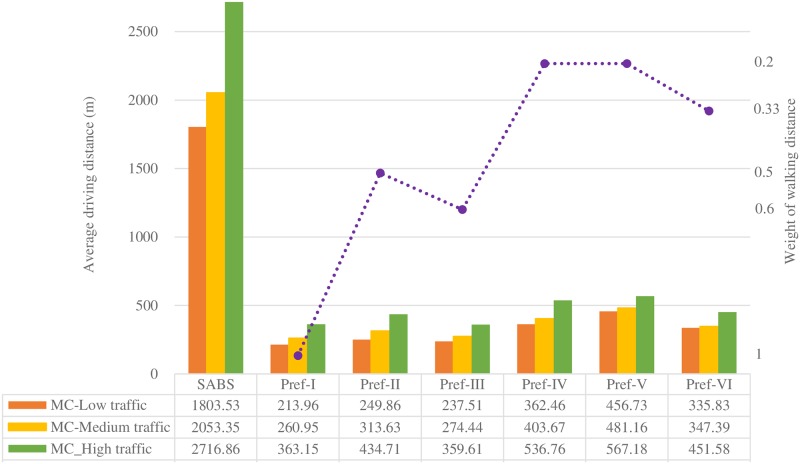
Average driving distance of the strategies with the Markov Chain-based availability definition.

**Fig 9:**
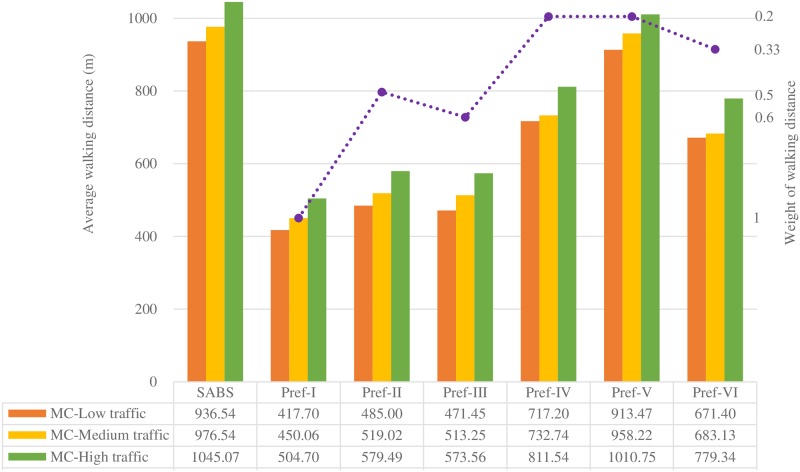
Average round trip walking distance of the strategies with the Markov Chain-based availability definition.

**Fig 10:**
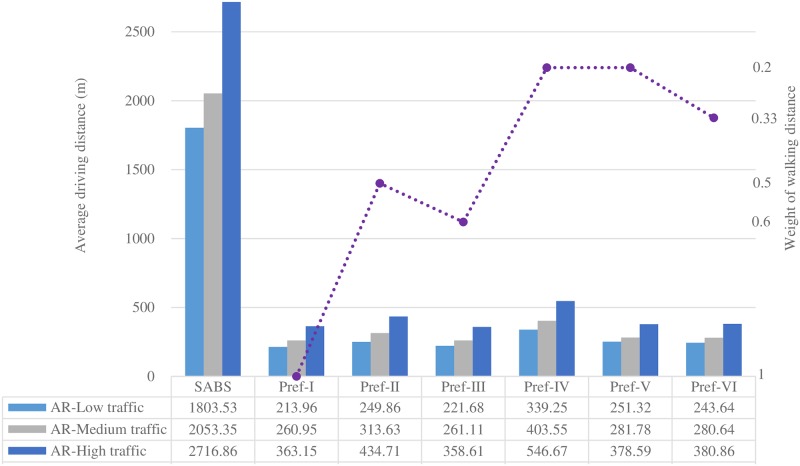
Average driving distance of the strategies with the arrival rate-based availability definition.

**Fig 11:**
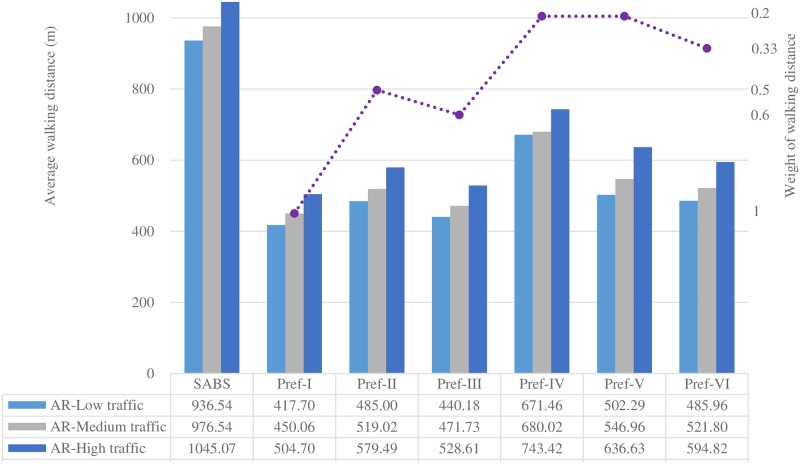
Average round trip walking distance of the strategies with the arrival rate-based availability definition.

**Fig 12:**
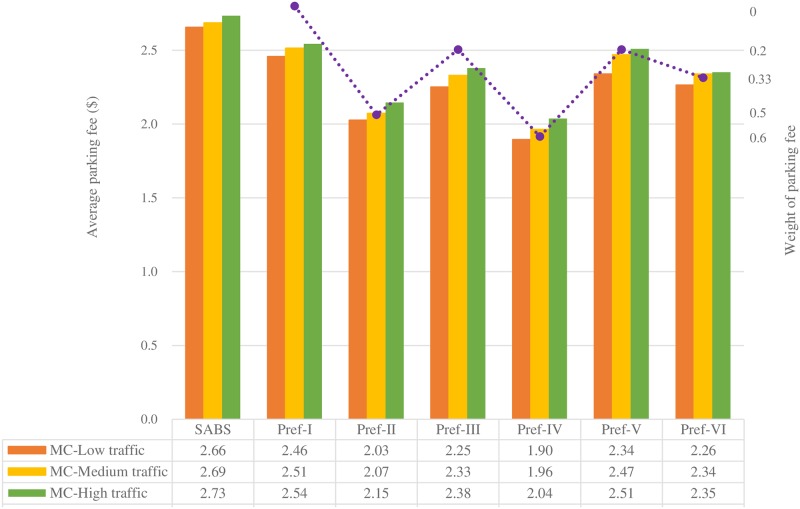
Average parking fee of the strategies with the Markov Chain-based availability attribute.

**Fig 13:**
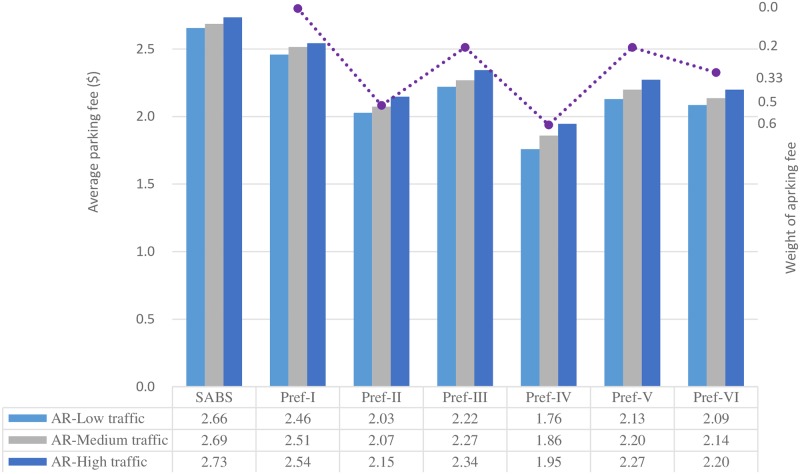
Average parking fee of the strategies with the arrival rate-based availability attribute.

Figs 7 to 13 present the simulation results for the SABS algorithm and the MADM-based algorithm with all preferences under low, medium, and high traffic. In these figures, the results of the SABS algorithm are always shown in the first category of the charts, and the results of preferences I to VI of the MADM-based algorithm are shown in the second to the last categories sequentially. For the MADM-based algorithm, the availability attribute of the parking facilities is involved in preferences III to VI, in which the weight corresponding to the availability attribute is not zero (see Table 3), and the way to define the availability attribute may influence the results of the MADM-based algorithm. So, in simulations, for every parking scenarios, both the arrival rate-based attribute and the Markov Chain-based attribute were used for the MADM-based algorithm respectively. In this way, for the parking scenarios with low, medium, and high traffic, we get six rows of results for the MADM-based algorithm:

1. When the arrival rate-based attribute is adopted, the results of the three traffic settings are shown in the AR-Low traffic row, the AR-Medium traffic row, and the AR-High traffic row correspondingly;
2. When the Markov Chain-based attribute is adopted, the results of the three traffic settings are correspondingly shown in the MC-Low traffic row, the MC-Medium traffic row, and the MC-High traffic row.

Notably, in Figs 7 to 13, the results of the first three categories (i.e., SABS, Pref-I, and Pref-II) are highly related to the traffic intensity, and not influenced by the availability attribute. So, no matter which availability attribute definition is adopted in the MADM-based algorithm, for every one of those three categories, it has the same results in the rows with the same traffic intensity. For example, in Fig 7, for the SABS category, when the traffic intensity is low, the results in both the AR-Low traffic row and the MC-Low traffic row are the same 1.81%.

#### Results of average parking failure rate

Fig 7 presents the average parking failure rate results of the SABS algorithm and the six preferences of the MADM-based algorithm, with both the arrival rate-based and the Markov Chain-based availability attributes and low, medium, and high traffic.

At first, we will analyze and compare the results of the SABS algorithm and the MADM-based algorithm with the Markov Chain-based availability attribute proposed in this paper. Then, analyze and compare the influence of the Markov Chain-based attribute and the arrival rate-based attribute on the results of the MADM-based algorithm.

When the traffic is very low, in most cases, any parking facility can have enough vacant parking spaces for incoming vehicles. For example, in simulations, when the average traffic intensities of the parking facilities are uniformly distributed in 0.1 to 0.7, with the expectation of 0.4, it was found that the average fail rates of the seven categories are almost the same 0%, and their results of other performance metrics, such as average walking distance and average parking fee, are very close to their results when the average traffic intensity is 0.7. So, in Fig 7, these results for the average traffic intensity 0.4 are not shown.

When the average traffic intensity of the parking facilities increases, for incoming vehicles, it generally becomes more and more difficult to find available parking spaces. This tendency is clearly shown in Fig 7. When the average traffic intensity is low (equal to 0.7), the overall average parking fail rates of all the categories are relatively low (not larger than 2.06%). As the average traffic intensity increases from low (equal to 0.7) to medium (equal to 1), except Pref-V and Pref-VI, all the other categories’ results increase notably. Furthermore, as the average traffic intensity increases from medium (equal to 1) to high (equal to 1.5), all categories’ average parking fail rates increase rapidly.

For the SABS algorithm, since the drivers always search for available parking facilities blindly, all the parking facilities can be regarded as having equal chances of being chosen by the drivers. For every row of results in Fig 7, the result of SABS falls within the range of the lowest parking failure rate to the highest one.

For Pref-I where the locations of the parking facilities are known and the walking distance is considered as an important decision factor, on condition that the locations of all the parking facilities and all the vehicles are uniformly distributed in the simulation area, just like SABS, all the parking facilities can also be regarded as having equal chances of being chosen by the drivers. This can explain why, in each row of Fig 7, the result of Pref-I is very close to that of SABS.

Besides the locations of the parking facilities considered in Pref-I, when parking fee is further considered in Pref-II, those low-cost parking facilities near the destinations of the drivers will be chosen. This will lead to higher competition of available parking spaces and higher average parking failure rate. So, in all the simulations for no matter low, medium, or high traffic parking environments, Pref-II always shows the highest average parking fail rates.

When the weight of parking fee decreases and the availability of vacant parking spaces is considered as in Pref-III, drivers may avoid those low-cost but fully-occupied nearby parking facilities. To some extent, this will relieve the competition of available parking spaces and result in a lower parking failure rate. As shown in Fig 7, in each row, compared with the average failure rate result of Pref-II, the result of Pref-III is always lower. Meanwhile, compared with SABS and Pref-I, in which the availability of vacant parking spaces is not taken into account, in Pref-III, the availability is an influencing factor. This will help drivers to reduce parking failure by avoiding those nearby but fully occupied parking facilities. So, as shown in Fig 7, the results of Pref-III are always lower than those of SABS and Pref-I.

Compared with SABS, Pref-I, and Pref-II that do not consider the availability of vacant parking spaces, not only Pref-III, other three preferences (i.e., Pref-IV, Pref-V, and Pref-VI) that consider the availability attribute of parking spaces can also produce lower parking failure rate.

Different from Pref-III in which the weight of *a*_*i*1_ (i.e., the walking distance attribute) is 0.6 and the weights of other two attributes are 0.2, in Pref-IV, the weight of *a*_*i*2_ (i.e., the parking fee attribute) increases from 0.2 to 0.6, and that of *a*_*i*1_ decreases from 0.6 to 0.2. This is equivalent to encourage drivers to expand their geological scope to find available parking spaces with lower parking fee. In this way, those cheaper parking spaces will be chosen by more drivers and lead to higher parking failure rate. So, in Fig 7, the results of Pref-IV are larger than those of Pref-III.

Now, turn to Pref-V, whose weight of the availability attribute is 0.6. As can be seen in Fig 7, in every row, by emphasizing on the availability of vacant parking spaces, Pref-V always has the lowest average parking failure rate result. This means Pref-V is the most effective one to find vacant parking spaces than SABS or the other preferences, which is in accordance with its weight assignment of the attributes. For example, in the rows of MC-Medium traffic and MC-High traffic where the Markov Chain-based availability attribute is used, the results of Pref-V are much lower than those of Pref-III and Pref-IV, needless to say SABS, Pref-I, and Pref-II.

At last, by assigning the same weight for all the three attributes, Pref-VI emphasizes on these attributes equally. Compared with Pref-V, this is equivalent to dilute the importance of the availability to the other attributes, leading to higher parking failure rate. So, in all simulations, the average parking failure rate results of Pref-VI are always higher than those of Pref-V. On the other hand, compared with SABS and other preferences except for Pref-V, Pref-VI is very effective to relieve the competition of parking resources by weighting all attributes equally. This can explain why in Fig 7 the average parking failure rate results of Pref-VI are always higher than those of Pref-V, but lower than all the others’.

After above analysis of the results of SABS and the six preferences one by one, now let’s investigate the effectiveness of the Markov Chain-based and the arrival rate-based availability attribute in the four availability-aware preferences (i.e., Pref-III, Pref-IV, Pref-V, and Pref-VI). Intuitively, compared with SABS, Pref-I, and Pref-II, for the same parking environment, since the availability attribute of parking spaces is considered in the four preferences, the drivers should gain more chances to find vacant parking spaces, and the average parking failure rate results of these four preferences should be lower than those of SABS, Pref-I, and Pref-II. This rule is validated in the rows of MC-Low traffic, MC-Medium traffic, and MC-High traffic in Fig 7, where the Markov Chain-based availability attribute is applied to the four preferences, and the average parking failure rate results of the four preferences are always lower than those of SABS, Pref-I, and Pref-II.

On the other hand, for the four availability-aware preferences, in a given parking environment, as the weight of the availability attribute increases, the drivers are more likely to choose those parking facilities with more available parking spaces, and the average parking failure rate results should decrease. This new rule is also validated in the rows of MC-Low traffic, MC-Medium traffic, and MC-High traffic in Fig 7, where the Markov Chain-based availability attribute is applied. For example, in the MC-High traffic row, the weight of the availability attribute in Pref-III, Pref-VI and Pref-V is 0.2, 0.33, and 0.6 increasingly, and the parking failure rate results are 18.95%, 12.96% and 11.46% decreasingly.

However, in the rows of AR-Low traffic, AR-Medium traffic, and AR-High traffic, where the arrival rate-based availability attribute is used for the four preferences, even though they can bring out lower parking failure rate results in some cases, they cannot guarantee to produce lower results in all cases. Take Pref-IV as an example, when the traffic intensity is medium, the result of Pref-IV in the AR-Medium traffic row is 11.23%, which is higher than the result 10.14% of SABS and 10.71% of Pref-I. When the traffic intensity increases to high, the performance of Pref-IV becomes worse: in the AR-High traffic row, the average parking failure rate result of Pref-IV increases to 27.36%, which is higher than the result 25.95% of SABS and 5.93% of Pref-I, and is very close to the result 27.47% of Pref-II, which is the highest result in all simulations.

Furthermore, as shown in Fig 7, for any one of these four availability-aware preferences, under no matter low, medium, or high traffic intensity, when the arrival rate-based availability attribute is applied, its average parking failure rate is always higher than that with the Markov Chain-based availability attribute. The differences are listed in Table 4. This tendency is more clear as the traffic intensity increases.

**Table 4:** Differences of the parking failure rate results of four availability-aware preferences with two different availability attributes.

	Pref-III	Pref-IV	Pref-V	Pref-VI
low traffic	0.07%	0.04%	0.13%	0.33%
medium traffic	2.49%	4.23%	3.68%	4.99%
high traffic	5.31%	6.99%	7.20%	9.38%

By comparing the results of the four preferences as above, it can be concluded that: compared with SABS, Pref-I, and Pref-II, when the Markov Chain-based availability attribute proposed in this paper is applied to the four availability-aware preferences for making parking guidance, the drivers will always have higher chances of finding available vacant parking spaces. Furthermore, as the weight of the availability attribute increases, the average parking failure rate will decrease.

Nevertheless, if the arrival rate-based availability attribute is applied, it may not be as effective as expected in many cases. Just as what is concluded by the authors in [15], it seems that *“the preferences used in the assignment do not have much effect on the parking failure rate”*. This conclusion pronounces the arrival rate-based availability attribute proposed in [15] is not effective enough to find available parking facilities.

When the arrival rate-based availability attribute proposed in [15] are applied to the availability-aware preferences, why it is not as effective to reduce the parking failure rate as the Markov Chain-based availability attribute defined in this paper? The root cause lies in the fact that the availability of parking spaces defined in [15] can not reflect the difficulty degree of finding available parking spaces properly. In this paper, we proposed a new queueing theory-based method to represent the availability of vacant parking spaces, which can remedy the problems found in [15]. The effectiveness of this method is justified by the simulation results shown in Fig 7. Further discussion about the ineffectiveness of the availability defined in [15] will be presented in Section Discussion.

Besides aforementioned driver-centric performance metrics, for every parking strategy or preference, the utilization of the parking facilities may also be concerned. For a given parking environment with specified parking facilities and vehicles, the average utilization of the facilities is inversely related to the average parking failure rate of the vehicles: a higher (lower) average parking failure rate means fewer (more) vehicles are accepted to park, leading to a lower (higher) utilization of the parking facility. So, based on above average parking failure rate results of SABS and all the preferences, the relative average utilization of the facilities can be inferred. For example, since Pref-V has the lowest parking failure rate of the vehicles, it will lead to the highest average utilization of the facilities. Generally speaking, for parking preferences considering the availability of vacant parking spaces, their average parking failure rate results are lower, thus leading to higher utilization of the parking facilities.

Considering that the arrival-rate based availability attribute is not as effective as the Markov Chain-based one to find available parking spaces, in following sections, when the average walking (driving) distance and the average parking fee are considered, we mainly focus on the results of the preferences with the Markov Chain-based availability attribute.

#### Results of average walking and driving distance

In simulations, the driving distance of a guided vehicle is defined as the length of the route from where the vehicle firstly sends out the parking requirement to the location of the parking facilities where the vehicle is accepted, and the walking distance is defined as the round trip length of the shortest path between the destination of the vehicle and the location of the parking facility where the vehicle is accepted. On condition that the locations of the parking facilities and the destinations of the drivers are known before parking, the drivers are assumed to choose the shortest driving or walking paths.

When the Markov Chain-based availability attribute is used, the results of the average driving distance and the average round trip walking distance of SABS and the six preferences are shown in Figs 8 and 9.

As can be seen in both figures, as the traffic intensity increases from low, medium to high, the average driving or walking distance of the strategies increase, in that as the traffic intensity increases, the parking failure rate of the vehicles increases, and the drivers have to try more times to find available parking facilities, and this will result in longer driving paths. On the other hand, as the traffic intensity increases, those parking facilities close to the destinations tend to be occupied more likely, and the drivers have to choose those farther ones. This will lead to longer average walking distance.

For SABS, since the locations of parking facilities are not known for drivers, and they always search for parking facilities blindly and choose the first one they meet, the walking distance between the location of the chosen facility to the destination is out of consideration. As can be seen, SABS’ average driving distance results are much longer than those of the six preferences, where the locations of the parking facilities are known and the shortest paths are always chosen. On the other hand, for SABS, when a driver arrives at his/her target parking facility, the locations of the facility is known, and the driver is assumed to choose the shortest path to walk to his/her destination. So, for SABS, its average walking distance results in Fig 9 are much shorter than its average driving distance results in Fig 8.

At the opposite extreme, for Pref-I, it is assumed that the locations of parking facilities are known and the weight of the walking distance attribute (*w*_1_ in Table 3) is as high as 1. So, the walking distance becomes the only criteria for choosing parking facilities: the parking facility with the shortest distance to the destination is always chosen at first. As expected, in every row of Fig 9, compared with the results of SABS and other preferences, the average walking distance result of Pref-I is always the shortest one.

For the six preferences from Pref-I to Pref-VI, in given parking environments, as the weight of the walking distance attribute is considered, their average walking distance results are always shorter than those of SABS. On the other hand, as the weight of the walking distance attribute decreases, their average walking distance results of the six preferences will increase. When the six preferences are sorted in a descending order of the weight of the walking distance attribute, the sorting result is Pref-I (*w*_1_ = 1), Pref-III (*w*_1_ = 0.6), Pref-II (*w*_1_ = 0.5), Pref-VI (*w*_1_ = 1/3), Pref-IV (*w*_1_ = 0.2), and Pref-V (*w*_1_ = 0.2). In each row of Fig 9, it can be found that this sorting result is in accordance with the six preferences’ ascending order of their average walking distance results.

For Pref-V, even though its weight of the walking distance attribute is the same 0.2 as that of Pref-IV, in every row of Fig 9, its average walking distance result is always higher than that of Pref-IV, and the highest among those of Pref-I to Pref-VI. It seems that Pref-V tends not to choose nearby parking facilities for drivers. This phenomenon can be explained as follows: in an extreme parking environment, assume all the drivers want to go the same destination, and there are several parking facilities scattered sparsely in this area. Since the walking distance attribute is used as a decision factor, those preferences with a larger weight of the attribute will prefer to choose nearer parking facilities. This will increase the occupancy of nearby facilities and decrease their availability, and stimulate Pref-V to choose those farther but more available parking facilities. So, finally, compared with other preferences, Pref-V always produces the highest average walking distance.

Figs 10 and 11 show the average driving and walking distance results of the strategies when the arrival rate-based availability attribute is used. Compared with the Markov Chain-based availability attribute, the arrival rate-based attribute tends to dilute the importance of the availability attribute. So, for the same preference with the same weight combinations of the attributes, the performance of the arrival rate-based attribute will lead to better results of the average walking(driving) distance and the average parking fee (see Fig 13). But, compared with the Markov Chain-based availability attribute, this can not be viewed as an advantage of the arrival rate-based attribute, in that this is incurred by the fact that the arrival rate-based attribute is less effective in finding available parking spaces than the Markov Chain-based one.

#### Results of average parking fee

Fig 12 presents the average parking fee results of the strategies in parking environments with various traffic intensities. The Markov Chain-based availability attribute is used for Pref-I to Pref-VI.

As the traffic intensity changes from low to high, for each parking strategy, generally speaking, the average parking fee increases. This can be explained by the fact that, as the traffic intensity increases, those parking facilities with lower cost may be fully occupied more frequently, thus drivers have to choose other parking facilities with higher cost, resulting in higher average parking fee.

In a given parking environment, according to the degree of emphasis on the parking fee attribute, different parking strategy or preference may have different parking fee result. As can be seen in Fig 13, for SABS and Pref-I, the parking fee attribute is not considered in choosing parking resources, thus their average parking fee results are high; On the contrary, for Pref-II to Pref-VI, their weights of the parking fee attribute are not less than 0.2, and their average parking fee results are relatively lower. Especially, the weight of the parking fee attribute in Pref-IV is as high as 0.6, thus this preference always focuses on finding parking resources with lower cost and finally has the lowest average parking fee results. For Pref-II, the weight of the parking fee attribute is also as high as 0.5, and this preference can also yield very good parking fee results, which are slightly higher than those of Pref-IV but notably lower than the others’. As to Pref-V, which pays more attention on finding available parking spaces, and less concerns on the parking fee, has the highest parking fee results among all parking fee attribute-aware preferences.

When the arrival rate-based availability definition is used for the preferences, their average parking fee results are shown in Fig 13. For SABS and Pref-I, since the parking fee attribute is not considered, their parking fee results are identical to those in Fig 12. However, for Pref-II to Pref-VI in which the parking fee attribute is used as a decision factor, their parking fee results are different from those in Fig 12. Just as what happened in Figs 10 and 11, the average parking fee results of Pref-II to Pref-VI in Fig 12 are lower than their counterparts in Fig 11. This can also be explained by the fact that the arrival rate-based availability definition dilutes the importance of the availability attribute.

## Discussion

In our simulations, as described above, compared with SABS, Pref-I, and Pref-II that neglect the influence of the availability attribute of parking spaces, those availability aware-preferences (i.e., Pref-III to Pref-VI) are more effective to reduce parking failure rate. Furthermore, it has been found that, in given parking environments, for every one of these four availability aware-preferences, compared with the arrival rate-based attribute, when the Markov Chain-based attribute is used, the preference will has lower average parking failure rate results, meaning that the Markov Chain-based attribute is more effective to represent the availability of parking spaces than the arrival rate based one proposed in [15].

In [15], it was shown that the preferences that consider the arrival rate-based availability attribute of vacant parking spaces are not effective on the reduction of parking failure rate. For example, preference VI in [15] is initially designed to emphasize on reduction parking failure by setting the weight of the availability of vacant parking spaces to be as large as 3. Instinctively, among all preferences, this preference should result in the lowest average parking failure rate. However, in all simulations with various traffic intensities, the average parking failure rate results of this preference have never been the lowest ones. Instead, another preference (i.e., preference III in [15]) that emphasizes on the walking distance always leads to the lowest average failure rate. These results are instinctively conflicting with the assignment of the preferences.

Why preference VI in [15] is not as effective as expected to reduce parking failure rate? Why in our simulations the arrival rate-based availability attribute is not as effective as the Markov Chain-based one? The root cause lies in the definition of the arrival rate-based availability. In [15], the degree of availability for the parking facility *j* is defined as $R_{ij}=\frac{T_{ij}/MTBA_{j}}{f_{j}}$, where *T*_*ij*_ is the estimated time for driving from current location to the target parking facility, *MTBA*_*j*_ the mean time between car arrivals of parking facility *j*, and *f*_*j*_ the number of currently vacant parking spaces. Compared with the queueing model adopted in our paper to represent the dynamical arriving and leaving process of vehicles in parking facilities, the model adopted in [15] is less effective: when estimate the number of available vacant parking spaces in a future time interval *T*_*ij*_, only the expected number of newly arriving vehicles in this period (i.e., *T*_*ij*_/*MTBA*_*j*_) is considered. Actually, in this period, currently parked vehicles may also leave, and those parking spaces occupied by those leaving vehicles will be available for incoming vehicles.

Furthermore, in [15], the lower value of *R*_*ij*_ is assumed to indicate that “it is more likely to find the free parking facility when a driver arrives at parking facility *j* since fewer cars are expected to come compared to the number of free parking facilities”. Unfortunately, for a driver about to arrive at parking facility *j* after *T*_*ij*_, the absolute number of possibly vacant parking spaces, rather than the ratio of the expected number of newly arriving vehicles to the number of currently vacant parking spaces as defined in *R*_*ij*_, will determine the degree of difficulty to find available parking spaces. For example, assume there are two parking facilities *j*1 and *j*2, and currently, their numbers of vacant parking spaces are 5 and 20 individually. Within *T*_*ij*_, assume the numbers of newly arrived vehicles (i.e., the values of *T*_*ij*_/*MTBA*_*j*_) for them are 2 and 8 individually. Then, according to the definition of *R*_*ij*_, their corresponding degrees of availability are 0.4(= 2/5) and 0.4(= 8/20). Since these two parking facilities have the same degree of availability, according to the definition of *R*_*ij*_, it seems that the driver should have the same failure rate to find one vacant parking space for the vehicle. However, this is far from the fact: the driver surely has more chances to find available parking space in parking facility *j*2 than in *j*1, in that the number of possibly vacant spaces in *j*2 is 12(= 20 − 8), and that of *j*1 is 3(= 5 − 2).

In our paper, we proposed new methods to represent the availability of vacant parking spaces, which can remedy the problems found in the availability definition adopted in [15]: on one hand, a queueing theory-based model is adopted to represent the dynamic process of parking facilities. Based on which, we can estimate the probabilities of all possible numbers of vacant parking spaces accurately in a given future time period for the parking facilities; On the other hand, the expected number of vacant parking spaces is used to represent the degree of difficulty to find available parking spaces. The effectiveness of these methods is justified by the simulation results.

## Conclusion

This study has introduced an MADM-based smart parking guidance algorithm by considering three representative decision factors and various preferences of drivers in parking environments. The decision factors include the walking distance between a facility and the destination of a driver, estimated parking cost, and the availability degree of vacant parking spaces, and the preferences emphasize on one or more factors by assigning different weights for them.

Besides walking distance and parking cost, the availability degree of vacant parking spaces is one determinant factor for choosing parking facilities, especially when the traffic intensity is very high and the probability of finding vacant parking spaces is small. In this study, to quantity the availability degree of vacant parking spaces in a parking facility, the facility is modeled as an M/M/c/c queueing system, and the probability of finding a given number vacant parking spaces in a future time interval is modeled as a Markov chain. In this way, on condition that the capacity of the facility, current number of vacant or occupied parking spaces, the arriving rate, the service rate, and the expected driving duration to the facility are known, the probability of finding any given number of vacant parking spaces in the facility is quantified. On the basis, the availability degree of this facility is defined as the expected number of vacant parking spaces.

To evaluate the performance of the proposed parking guidance algorithm, experiments were carried out in simulated parking environments. For the MADM-based algorithm, both the Markov Chain-based availability attribute and the arrival rate-based availability attribute proposed in [15] were considered for those availability-aware preferences, and their performance was investigated and compared with the base self-avoiding blind search algorithm in terms of the average parking failure rate, the average driving and walking distance, and the average parking cost.

Experimental results show that every preference with the Markov Chain-based availability attribute can always choose the appropriate parking facility to satisfy the emphasized assignment, indicating that the proposed parking guidance framework and the MADM-based algorithm are effective to help drivers with various preferences find proper parking resources. Furthermore, it has also been observed that the Markov Chain-based attribute is more effective to represent the availability of parking spaces than the arrival rate-based attribute proposed in [15].

So far as the authors know, compared with existing parking guidance algorithms that considered the availability of vacant parking spaces but proved to be not so effective, up to now, the proposed algorithm in this study is the only one that was proved to be effective. The algorithm itself, especially the methods to model and estimate the number of vacant parking spaces in a given time period, can be imported into real parking guidance systems or other parking guidance algorithms.

## Appendix: Steady state probability

For parking facility *P*_*i*_, *i* = 1, 2, …, *n*, with capacity *c*_*i*_, arrival rate λ_*i*_, and service rate *μ*_*i*_, the steady state probability of finding *k* occupied parking spaces can be obtained as below [24]:

$$\begin{array}{c} p_{k}=\frac{{\left(\lambda_{i}/\mu_{i}\right)}^{k}}{k!}/\left(\sum_{i=0}^{c_{i}}\frac{{\left(\lambda_{i}/\mu_{i}\right)}^{i}}{i!}\right),\left(0\leq k\leq c_{i}\right) \end{array}$$ (10)

According to the probability for each value of *k*, we can choose one value ranging from 0 to *c*_*i*_ as the initial number of vehicles that are assumed have already been parked in *P*_*i*_ at the beginning of the simulation. Then, such number of vehicles will be generated and added into the parking facility. Since the vehicles in the same parking facility will follow the same exponential distribution, and an exponentially distributed random variable *T* has the memory-less property, i.e.,

$$\begin{array}{c} Pr(T>s+t|T>s)=Pr(T>t),\forall s,t\geq 0, \end{array}$$ (11)

where *T* can be interpreted as the service time of an initially parked vehicle ranging from its arrival time to its leaving time. For any parking facility, we assume the service times of these vehicles that are assumed have been parked in the parking facility at the start time of the simulation will follow the same exponential distribution as those which arrive after the beginning of the simulation.

In Formula 10, when the number of spaces *c* is large, terms like *c*! may cause computational problems on computers if they are implemented directly. For example, when *c* is larger than 200, the value of *p*_*k*_ is often returned as a *NaN(not a number)* in Matlab. To handle this problem in simulation, once the value of *p*_*k*_ is returned as *NaN*, another alternative method is used to estimate the number of initially parked vehicles in parking facility *P*_*i*_. Assume the traffic intensity of *P*_*i*_ is *ρ*_*i*_, define *ρ*_*i*1_ = *max*(0, *ρ*_*i*_ − 0.2), and *ρ*_*i*2_ = *max*(1, *ρ*_*i*_ + 0.2), let the number of initially parked vehicles be *fix*(*c*_*i*_ * *U*(*ρ*_*i*1_, *ρ*_*i*2_)), where *fix*() is used to round a variable to the nearest integer towards zero, and *U*(*ρ*_*i*1_, *ρ*_*i*2_) is used to generate a random variable uniformly distributed within *ρ*_*i*1_ and *ρ*_*i*2_.

## Supporting information

S1 File Simulation files used in the simulations and results section. (RAR)
